# Silencing of Neuropilins and GIPC1 in pancreatic ductal adenocarcinoma exerts multiple cellular and molecular antitumor effects

**DOI:** 10.1038/s41598-019-51881-8

**Published:** 2019-10-29

**Authors:** Hannes Borchardt, Alexander Schulz, Kaustubh Datta, Michael H. Muders, Achim Aigner

**Affiliations:** 1 Rudolf-Boehm-Institute for Pharmacology and Toxicology, Clinical Pharmacology, Medical Faculty, University of Leipzig, Leipzig, Germany; 2 Institute of Pathology, University Hospital Carl Gustav Carus, University of Technology, Dresden, Germany; 3 Department of Biochemistry, University of Nebraska Medical Center, Omaha, Nebraska USA; 4 Rudolf-Becker-Laboratory for Prostate Cancer Research, Institute of Pathology, University of Bonn Medical Center, Bonn, Germany

**Keywords:** Cancer, Drug discovery

## Abstract

Pancreatic ductal adenocarcinoma (PDAC) is a leading cause of cancer mortality, with new treatment options urgently needed. Neuropilins-1/-2 (NRP1, NRP2) are receptors for semaphorins and angiogenic growth factors, while the GAIP interacting protein C-terminus 1 (GIPC1, aka Synectin) interacts with the neuropilins. They are overexpressed in PDAC and associated with poor survival as well as tumor-promoting activities. Thus, neuropilin and/or GIPC1 silencing may inhibit PDAC growth. In this study, we directly compare the various tumor-inhibitory effects of transient RNAi-mediated depletion of NRP1, NRP2 and GIPC1, alone or in combination, in a set of cell lines with different expression levels. Inhibition of anchorage-dependent and –independent proliferation, colony formation and cell migration, alterations of 3D-spheroid size and shape as well as retardation of cell cycle and induction of apoptosis have been analyzed and found to vary between cell lines. The observed effects are independent of initial expression levels. Knocking down NRP1, NRP2, and GIPC1 alone demonstrates significant effects. Only small additive effects upon combined knockdown and no counter-upregulation of the respective other genes could be detected. Making the study more translational, we show that systemic treatment of PDAC xenograft-bearing mice with polymeric nanoparticles for delivery of specific siRNAs results in tumor inhibition, reduces proliferation, and induces apoptosis. In conclusion, NRP and GIPC1 inhibition emerges as a promising avenue in PDAC treatment due to pleiotropic tumor-inhibitory effects.

## Introduction

Pancreatic cancer is one of the leading causes of cancer mortality in developed countries. There are two main tumor types of pancreatic cancer: ductal adenocarcinoma of the pancreas (PDAC) and pancreatic endocrine tumors. Ductal adenocarcinoma accounts for 85% of cases, with most of them being detected in a late stage. In terms of incidence, it remains the 5^th^ cause of cancer related deaths. Because of limited efficacy of available therapies, the five year survival rate in PDAC is less than 5%^1,2^. Consequently, new treatment options based on novel targets and strategies are urgently needed. The targeting of growth promoting receptor molecules by RNAi-mediated gene knockdown, based on siRNA formulations for their delivery, might be a promising approach.

Regulators of the neuropilin axis are potential targets for this RNAi-based therapy. Neuropilins are non-tyrosine kinase receptors for semaphorins and angiogenic growth factors that play a role in axonal guidance and angiogenesis^3–5^. Both neuropilins, i.e., neuropilin-1 (NRP1) and neuropilin-2 (NRP2), are overexpressed in various cancer types and their expressions have been correlated with tumor progression and poor prognosis^3^. Importantly, several reports described significant overexpression of NRP1 and NRP2 in PDAC, which were associated with poor survival^6–15^. NRP1 is involved in oncogenic processes like EMT^16^, cell invasion^8^, chemoresistance^17^ and angiogenesis^10^. We previously showed that NRP2 regulates endosomal trafficking and autophagy as well as EGFR signaling in PDAC^18,19^ and that NRP2 blockage promoted tumor-inhibition^14^.

Neuropilins have very short intracytoplasmic domains that are dependent on scaffold proteins to transduce biological signals. One of these proteins is the GAIP interacting protein C-terminus 1 (GIPC1, also known as Synectin). GIPC1 interacts with the neuropilins via its PDZ domain and their three C-terminal amino acids SEA, respectively^20–23^. GIPC1 is also highly expressed in pancreatic cancer^24^. Because of its interaction with the PDZ domain of cell surface receptors and signaling molecules, it is a central player in various signaling pathways, modulating important cell functions like migration and proliferation. Stable knockdown or targeting the PDZ domain using small peptide inhibitors has been shown to significantly inhibit pancreatic cancer growth^25^. Thus, silencing of neuropilins or GIPC1 has the potential of reducing pancreatic cancer growth *in vitro* and *in vivo*^13^.

In this study, we have compared the various tumor-inhibitory effects following transient RNAi-mediated depletion of NRP1, NRP2 or GIPC1 either alone or in combination, in PDAC cell lines with different expression levels. Pushing the system further towards therapy, we employ polymeric nanoparticle-based siRNA delivery to inhibit NRPs and GIPC1 for the treatment of PDAC xenograft-bearing mice.

## Methods

### Cell lines and culture conditions

Pancreatic cancer cell lines (AsPC1, CaPan1, Colo357, Hs766t, IMIM-PC1, MiaPaca2, Panc1, Panc89, PaTu 8988t) were obtained from the American Type Culture Collection (ATCC, Manassas, VA). Cells were cultured under standard conditions (37 °C, 5% CO_2_) in RPMI 1640 Medium (Sigma, Taufkirchen, Germany) supplemented with 10% heat-inactivated fetal bovine serum (FBS Superior, Biochrom, Berlin, Germany), unless stated otherwise.

### Transient transfection

SiRNAs were purchased from Ambion Life Technolgies/Thermo Fisher Scientific (Waltham, MA) and from MWG Eurofins (Ebersberg, Germany). Sequences are provided in Suppl. Table 1. As negative control (siCtrl), siRNA against luciferase (pGL3) was used. Cells were seeded one day prior to transfection with 5–20 nM siRNA at appropriate cell densities, dependent on the assay, the plate size and the cell line, and were kept under standard conditions. INTERFERin^TM^ (Polyplus, Illkirch, France) was used as transfection reagent according to the manufacturer’s protocol. In the case of double knockdown experiments, identical specific as well as total siRNA amounts were used for better comparison between single and combined treatment, i.e., in the case of single knockdown the specific siRNA was mixed with the same amount of negative control siRNA.

### RNA preparation and quantitative RT-PCR for mRNA detection

Total RNA from cultivated tumor cells was isolated by using the EXTRAzol reagent (Blirt, Gdansk, Poland) according to the manufacturer’s protocol. For cDNA synthesis, the RevertAid RT Kit was used (Thermo Fisher) and semi quantitative PCR was performed using the StepOnePlus™ Real Time System and PerfeCTa^®^ SYBR^®^ Green FastMix^®^, ROX (QuantaBio, Hilden, Germany) according to the manufacturer’s protocol. The cDNA was used as a template for semi-quantitative real-time PCR under following conditions: 95 °C for 15 sec followed by 45 cycles comprising 95 °C for 10 sec, 55 °C for 10 sec and 72 °C for 10 sec. Levels of mRNA were determined by using the formula 2^-(CP(target)-CP(hk))^ with CP = cycle number at the crossing point. To normalize for different cDNA amounts, the housekeeping genes β-actin, GAPDH and RPLP0 were run in parallel. All primer sequences are given in Suppl. Table 2.

### Soft agar assay, scratch assay, spheroid and colony formation

To assess anchorage-independent growth and proliferation, soft agar assays were performed as described in the Suppl. Information. For spheroid formation assay, 5 × 10^3^ cells in 200 µl cell culture medium were seeded in ultra-low attachment 96-well plates (Brand Scientific, Wertheim, Germany). Spheroid formation was documented over several days under the microscope, and from microscopic pictures areas or volumes were measured using ImageJ. Colony formation and cell migration (scratch assay) were assessed as described in the Suppl. Information.

### Caspase assay, annexin V staining and cell cycle analysis

A commercially available bioluminescent caspase-3/7 assay (Caspase-Glo-3/7 assay; Promega) was used according to the manufacturer’s protocol. The assay was performed in white-wall 96-well plates as described in the Suppl. Information. To quantitate viable, dead, apoptotic and necrotic cells, a flow cytometry-based fluorescein isothiocyanate (FITC)-Annexin assay (Invitrogen, Carlsbad, CA) was performed (see Suppl. Information). For cell cycle analysis, cells were treated with nocodazole and further processed as described in the Suppl. Information.

### PEI complexation of siRNAs

PEI/siRNA nanoparticles were prepared as described previously^26^. Briefly, 10 µg siRNA was dissolved in 75 µL HN-buffer (10 mM HEPES/150 mM NaCl, pH 7.4) and incubated for 10 min at room temperature. In a second vial, 8.6 µL of PEI F25-LMW (6.1 µg/mL) was diluted in 75 µL of the same buffer. After 10 min, the mixture was pipetted to the siRNA solution, vortexed and incubated for 30 min at room temperature. When upscaling the complex formation, the complexes were aliquoted and stored at −80 °C. Prior to use, complexes were thawed, briefly vortexed and incubated at room temperature for 30 min.

### PEI F25-LMW/siRNA treatment in mouse xenograft models

All mouse experiments were performed strictly according to the national regulations of animal welfare. They were reviewed by the local committee on animal welfare (Leipzig committee on animal welfare, according to §15 Tierschutzgesetz) and finally approved the local authorities (Landesdirektion Sachsen, Referat für Veterinärwesen und Lebensmittelüberwachung, Germany).

6–8 weeks old athymic nude mice (Crl:CD1-Foxn1nu, Charles River Laboratories, Sulzfeld, Germany) were kept in cages with rodent chow (ssniff, Soest, Germany) and water available *ad libitum*. 5 × 10^6^ Colo357 cells in 150 µL PBS were injected subcutaneously into both mouse flanks. When the tumors were established, mice were randomized into three treatment (PEI/siGIPC1, PEI/siNRP1, PEI/siNRP2) and two control groups (untreated, PEI/siCtrl), with n = 10–12 mice per group. Mice were treated 3x/week by intraperitoneal injection of PEI F25-LMW/siRNA complexes containing 10 µg siRNA in 150 µL HN buffer. Tumor volumes were measured at the same time points. Upon termination of the experiment, mice were sacrificed and tumors were immediately removed, snap-frozen for RNA analysis and protein preparation, or fixed in 4% paraformaldehyde prior to paraffin embedding.

### Western blotting

Cells in 6-well plates and frozen tumor samples were lysed by adding appropriate volumes of RIPA buffer (Thermo Scientific) supplemented with complete inhibitor (Roche), Halt protease inhibitor and Halt phosphatase inhibitor (both from Thermo Scientific). The lysate was collected in a 1.5 mL tube and further processed as described in the Suppl. Information.

### Immunohistochemistry

The protein expression of NRP1, NRP2 and GIPC1 was analyzed by immunohistochemistry (IHC) using commercially available antibodies (Suppl. Table 3). Staining for GIPC1 and NRP2 was performed on 2 µm sections as described previously (^27^, Borkowetz *et al*., Int J Cancer, in press) using the immunoperoxidase-based universal VECTASTAIN Elite ABC HRP kit (Vector laboratories, Burlingham, CA) 3,3’-Diaminobenzidine tetrahydrochloride as well as 3-amino-9-ethylcarbazole (AEC) (both Sigma-Aldrich, Steinheim, Germany). In general, staining was performed using a staining machine (LabVision, ThermoScientific, Karlsruhe, Germany). The stainings were evaluated by an experienced board certified surgical pathologist (MHM) as well as a researcher (AS) and scored as 1- low, 2- middle, 3- high, using an Olympus BX45 microscope. Photos were taken using a DP22-Olympus microscope camera. Cleaved Caspase-3 (perinuclear staining) and Ki67 (nuclear staining) were evaluated by counting the positive cells in hot spots of the slides in 10 high power fields and by calculating the percentage positive cells.

### Statistical analyses

All experiments were performed at least in triplicates unless indicated otherwise. Results were analyzed for differences between siCtrl- and the specific siRNAs using Student’s t-test or Wilcoxon-Mann-Whitney test, with *p < 0.05, **p < 0.01, ***p < 0.001 and # = n.s.

## Results

### Expression and siRNA-mediated knockdown of Neuropilins and GIPC1 in various pancreatic carcinoma cell lines

**Figure 1:**
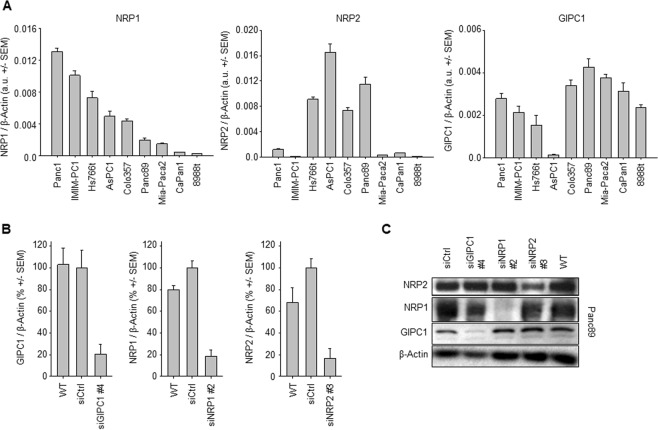
(**A**) Expression levels of Neuropilins 1 and 2, and of GIPC1, in various pancreatic carcinoma cell lines. (**B**) Knockdown of target gene mRNA mediated by optimal siRNAs, as determined in AsPC1 cells. (**C**) Western blots demonstrating the reduction of target gene protein levels in Panc89 cells.

Expression levels of NRP1, NRP2 and GIPC1 in nine pancreatic carcinoma cell lines were tested by RT-qPCR and revealed major differences (Fig. 1A). Panc1 and IMIM-PC1 cells showed highest NRP1 mRNA levels, being more than 30-fold higher than CaPan1 or PaTu 8988t cells (Fig. 1A, left). NRP2 mRNA was highest in AsPC1 and Panc89 and low in IMIM-PC1 cells (Fig. 1A, center). Similar results were found in PaTu 8988t cells, which showed, however, intermediate GIPC1 expression. GIPC1 mRNA levels showed less variation among the cell lines (~2.5 fold changes), except for AsPC1 cells with profoundly lower values (Fig. 1A, right). For all three genes, transcript abundance was independent of cell confluency and presence/absence of fetal bovine serum in the cell culture media, as demonstrated in Colo357 cells (Suppl. Fig. 1A). This cell line, which shows intermediate levels of all transcripts, was also used for testing three different siRNAs per target gene. Among the siRNAs, significant inhibition was observed with siGIPC1 #4, siNRP1 #2 and siRNP2 #3 (Suppl. Fig. 1B). These siRNAs were therefore selected for further experiments. Knockdown efficacy was confirmed in AsPC1 cells, with >80% reduction of target gene expression (Fig. 1B) that was similar to Colo357 cells (Suppl. Fig. 1C). Significant reduction of the target genes following siRNA transfection was also confirmed on the protein level by western blot (Fig. 1C and Suppl. Fig. 1D). Notably, knockdown efficacies were largely independent of initial target gene expression levels, and the reduction of the respective target gene did not affect the other family members. More specifically, no off-target effects in terms of the reduction of the other targets were observed (Fig. 1C and Suppl. Fig. 1D), and the prolonged cultivation of the cells after knocking down of a given target did not lead to the counter-upregulation of the other family members (not shown). Based on their different expression levels, and to cover a wider range of expression levels, the cell lines PaTu 8988t, Colo357, AsPC1, Panc1 and Panc89 were selected for further experiments.

### Effects of NRP1/2 and GIPC1 knockdown on cell viability and proliferation

**Figure 2:**
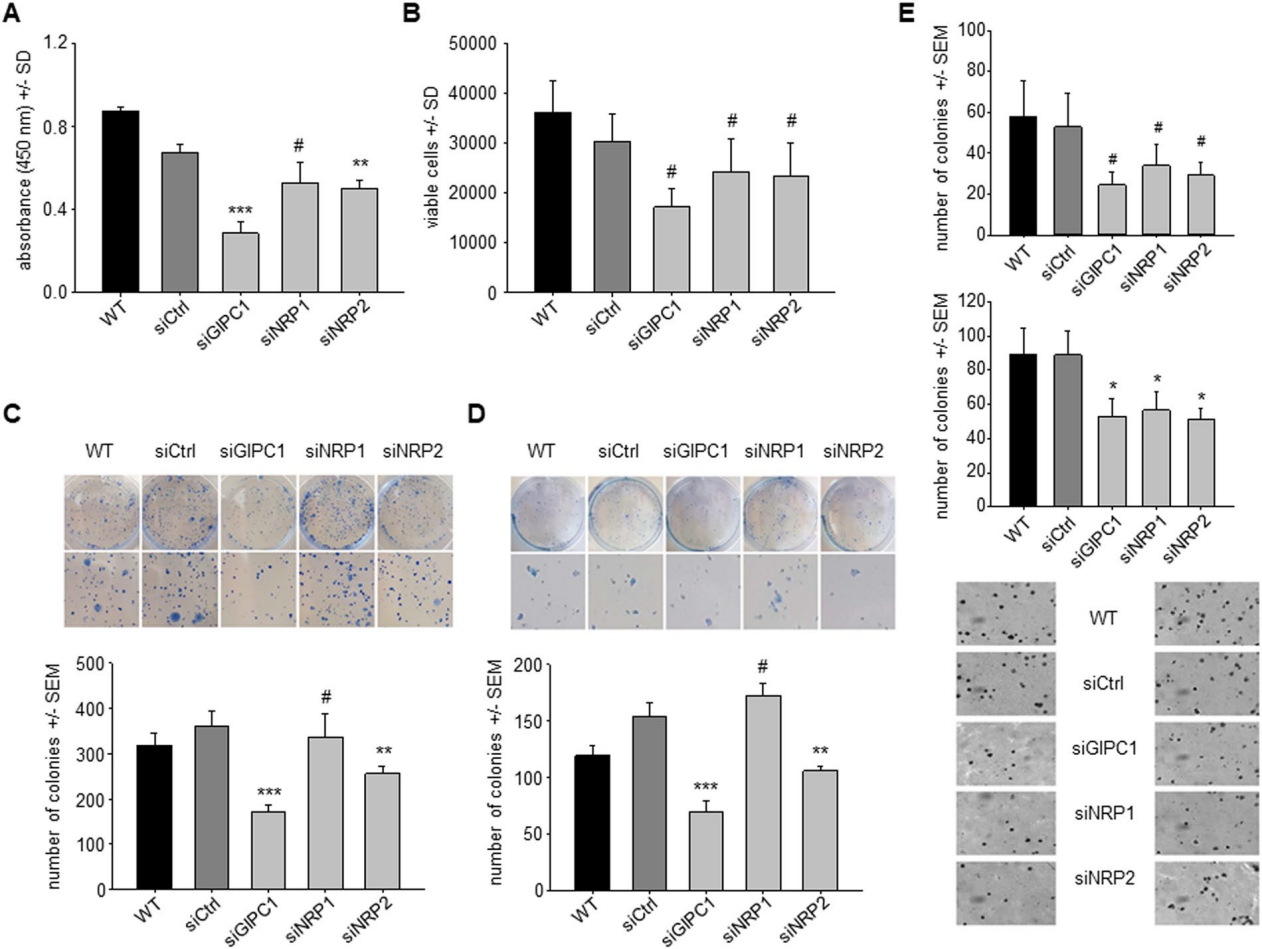
(**A**) Anchorage-dependent proliferation assay in PaTu 8988t cells, based on WST-1 quantitation. (**B**) Quantitation of Colo357 cells in anchorage-dependent proliferation. (**C**,**D**) Colony formation assays in Colo357 cells (**C**) and Panc89 cells (**D**). (**E**) Soft agar assay (Colo357 cells).

The transient siRNA-mediated knockdown of GIPC1 in PaTu 8988t cells showed a >50% reduction in anchorage-dependent proliferation, as determined by WST-1 after 96 h (Fig. 2A). In contrast, only a moderate ~20% inhibition was observed in the case of siNRP1 or siNRP2. While this could be in part explained by the very low NRP1 and NRP2 expression levels in this cell line, very similar data were also obtained in Colo357 cells (Fig. 2B) which show more profound expression of all three target genes (see Fig. 1A). Similar results were observed in colony formation assays in different cell lines, with only siGIPC1 showing a ~50% inhibition as compared to siCtrl (Fig. 2C,D and Suppl. Fig. 2A). In contrast, neuropilin knockdown showed overall little (NRP1) or no (NRP2) effect in all three cell lines. Concomitantly, caspase-3/7 activation was observed only upon siGIPC1 transfection (Suppl. Fig. 2B). In contrast, anchorage-independent proliferation was inhibited after the knockdown of each of the three target genes, as determined by soft agar assays (Fig. 2E). The comparison of the Colo357 results from Fig. 2B–D also indicates that measuring anchorage-dependent proliferation may rather underestimate anti-proliferative effects, while anchorage-independent soft agars represent more closely the *in vivo* situation. Likewise, in AsPC1 cells a ~40% inhibition of soft agar colony growth was observed for all three genes. This is particularly noteworthy since in this cell line mRNA levels were found very low for GIPC1 and high for NRP2 (see Fig. 1A). Taken together, this indicates that inhibitory effects on tumor cells upon GIPC1 or neuropilin knockdown are dependent on the assay rather than the cell line and the endogenous expression levels of the target genes. The expression levels of NRPs and GIPC1 considerably vary between the cell lines and do not seem to be the major determinant of the various cellular functions in the steady state condition.

### Alterations in cell migration and spheroid shape/formation

**Figure 3:**
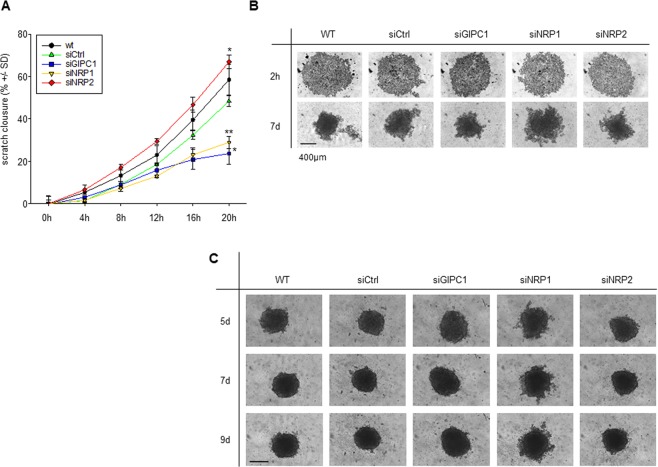
(**A**) Quantitation of time-dependent scratch closure in a wound healing assay (Panc89 cells). (**B**,**C**) Spheroid assays in Panc-1 cells, with transfection 1 d prior to spheroid seeding (**B**) or parallel transfection during spheroid seeding (**C**).

Similar to the above result, knocking down both GIPC1 and NRP1 inhibited migration of PDAC as observed in scratch assay. In contrast, cell migration was enhanced upon NRP2 knockdown when compared to control (negative control siRNA transfected or untreated cells) (Fig. 3A and Suppl. Fig. 2C). Spheroid formation of cells pre-transfected with siGIPC1 or siNRP2 was inhibited although not significantly after 7 days of culture (Fig. 3B). In contrast, NRP1 and GIPC1 knockdown led to even larger spheroids. Counting the cells after trypsinization of the spheroids, however, revealed no increase in cell numbers, indicating that the larger spheroid size was based on lower spheroid density rather than increased proliferation. This was also seen when cells were transfected during spheroid formation. Despite the later onset of the knockdown only after spheroid formation, a less dense structure and an uneven spheroid surface was observed in the case of siNRP1 (Fig. 3C).

### Induction of cell cycle alterations and cell death

**Figure 4:**
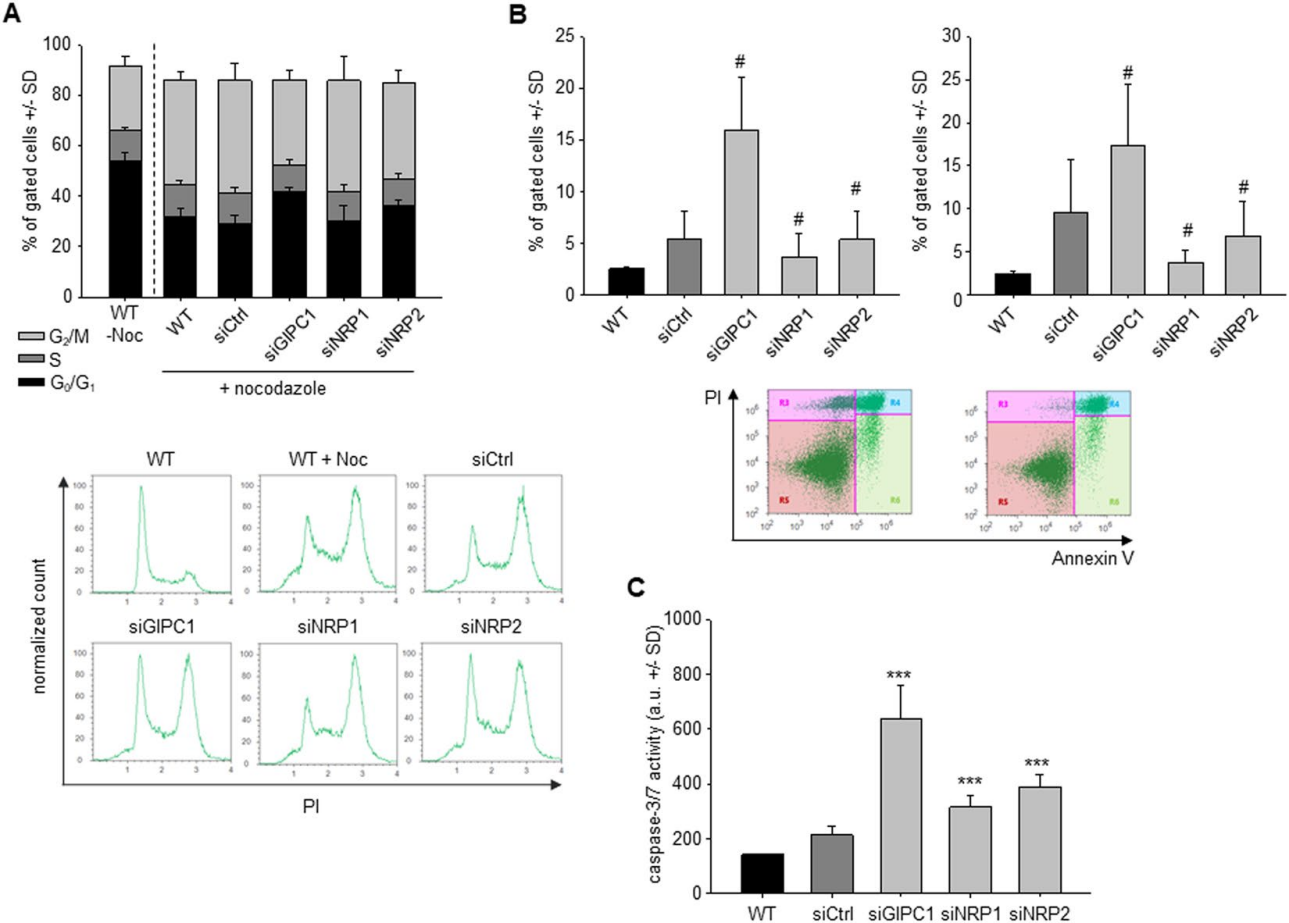
(**A**) Determination of cell cycle distribution in PaTu 8988t cells at 14 h after introducing a nocodazole block. (**B**) Flow cytometry-based determination of apoptotic (left) and necrotic (right) PaTu 8988t cells. Lower panel: representative examples of cell populations in the gated areas (left: siCtrl, right: siGIPC1). (**C**) Caspase activation in PaTu 8988t cells 96 h after knockdown.

The knockdown of GIPC1 led to a rather profound inhibition of cell cycle, as determined by flow cytometry. PaTu 8988t cells were initially transfected with siRNAs targeting NRPs or GIPC1. Upon onset of the specific knockdown, G2/M cell cycle arrest of the transfected cells was induced by nocodazole. The cell cycle distribution was evaluated after another 14 h. The lower proportion of cells reaching the G2/M block in the case of siGIPC1 indicates slower cell cycle progression (Fig. 4A). A slight cell cycle inhibition was also observed upon siNRP2 transfection, while NRP1 knockdown did not have any effect. GIPC1 knockdown also led to increased cell death in PaTu 8988t cells. Flow cytometry analysis revealed ~3-fold increase in apoptotic cells (Fig. 4B, left) as well as an increase in necrotic cells (Fig. 4B, right). Essentially no effect on apoptosis was observed following the depletion of neuropilins. The activity of caspases-3/7 was also ~3-fold higher 96 h after GIPC1 knockdown, but only slightly increased upon siNRP1 or siNRP2 transfection (Fig. 4C). The effects of NRPs and GIPC1 on cell death, however, were dependent on the cell line. In AsPC1 cells, siGIPC1 knockdown did not lead to an induction of apoptosis. While this could be explained by the very low endogenous GIPC1 levels in this cell line, no increase in apoptosis following the knockdown of NRP2 (which is expressed in a significant level) suggests no involvement of NRP2 in cell survival in this cell line (Suppl. Fig. 3A, left). In contrast, Colo357 cells showed a profound induction of apoptosis upon siGIPC1 transfection as seen also in PaTu 8988t cells, and a slight but statistically significant increase in apoptosis following NRP2 depletion (Suppl. Fig. 3A, right). The apoptosis and secondary necrosis of these cells following the depletion of NRPs and GIPC1 was further confirmed by LDH release assay (Suppl. Fig. 3B). Here, the siNRP2 effect was more prominent while siNRP1 again did not affect the cell survival of PDAC.

### Tumor cell inhibitory effects upon combination knockdown

**Figure 5:**
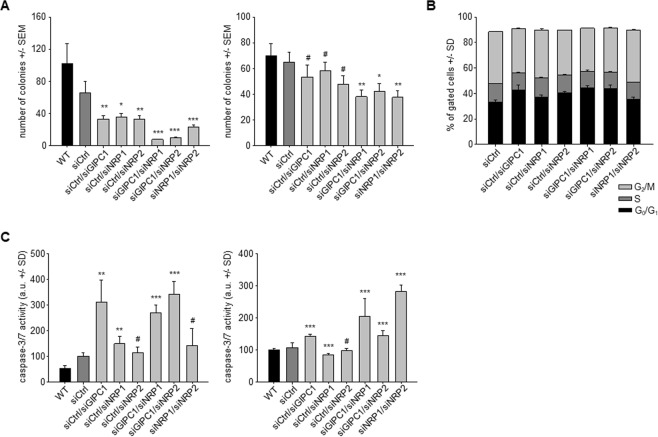
Effects of combined knockdown. (**A**) Soft agar assays on Colo357 (left) and AsPC1 cells (right). (**B**) Determination of cell cycle distribution in PaTu 8988t cells at 16 h after introducing a nocodazole block. (**C**) Caspase activation in PaTu 8988t (left) and Panc1 cells (right) 72 h after knockdown.

Based on their biological roles, additive or even synergistic effects might be expected upon combining the knockdown of more than one target gene. Indeed, soft agar assays in Colo357 cells revealed an almost complete inhibition of colony formation upon double knockdown of GIPC1 and either NRP1 or NRP2, thus profoundly enhancing the already ~50% inhibition of the single siRNA transfections (Fig. 5A, left). No major increase in inhibition, however, was observed upon combining siNRP1 and siNRP2. In other assays, combination effects were less profound. Anchorage-dependent proliferation assays in the same cell line revealed minor benefit from siRNA combinations (Suppl. Fig. 3C), while the apoptosis assay showed an increase in caspase-3/7 activity mainly upon siNRP2 knockdown, independent of single or double transfection (Suppl. Fig. 3D). Likewise, alterations in cell cycle (Fig. 5B) or induction of apoptosis (Fig. 5C, left) in PaTu 8988t cells was predominantly driven by GIPC1 knockdown, with no additional effect of siNRP1 or siNRP2. One exception was apoptosis in Panc1 cells, where simultaneous knockdown of NRP1 and NRP2 led to markedly increased caspase-3/7 activation (Fig. 5C, right). This was somewhat unexpected since NRP2 is only weakly expressed in this cell line, again indicating that expression level is a poor predictor of biological relevance of these target genes.

### Therapeutic inhibition of tumor xenograft growth upon treatment with nanoparticle-formulated siRNAs

**Figure 6:**
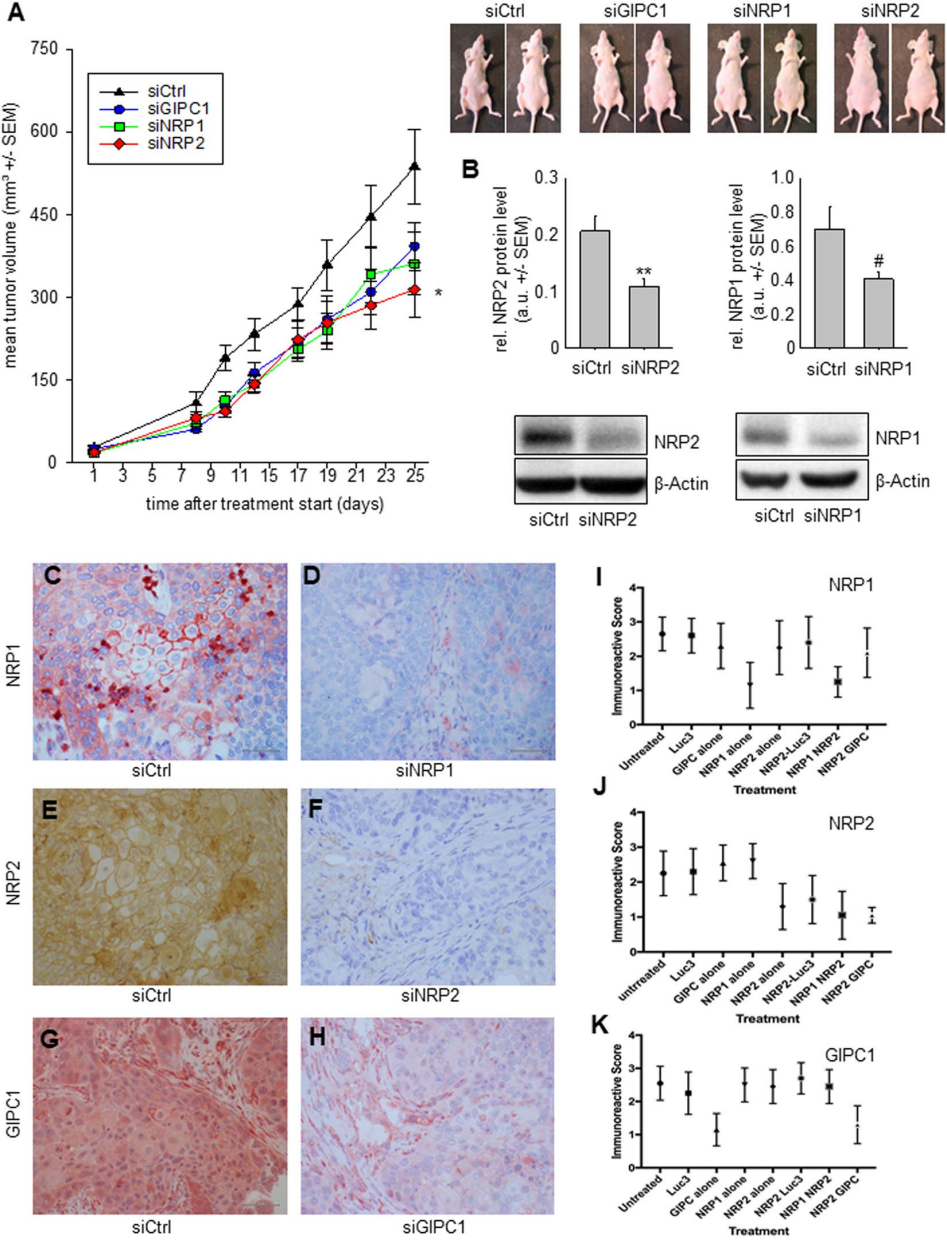
Therapy study in s.c. xenograft-bearing mice using nanoparticle-formulated siRNAs. (**A**) Tumor growth curves of Colo357 xenografts upon treatment with PEI-complexed specific or non-specific siRNAs. Right panel: representative examples of mice upon termination of the experiment. (**B**) Quantitation of NRP2 (left) or NRP1 protein levels (right) in tumor xenografts after explantation. Lower panel: representative Western blots of tumor lysates from tumor xenografts treated as indicated. (**C–H**) Immunohistochemistry of transplanted tumors, stained with antibodies against the silenced proteins. Representative tissue pieces of tumor xenografts from mice treated with specific siRNA (right panels) vs. negative control treatments (left panels) are shown. Tissues are stained for NRP1 (**C,D**), NRP2 (**E,F**) and GIPC1 (**G,H**). Bar: 2 µm. (**I–K**) Graphs illustrating the quantitation of immunoreactive staining. Means of the immunoreactive score of the specific staining is given for all treatment groups, with tissues stained for NRP1 (**I**), NRP2 (**J**) and GIPC1 (**K**).

To test tumor-inhibitory effects of siRNA-mediated knockdown of GIPC1, NRP1 and NRP2 more rigorously in an *in vivo* model, we subcutaneously transplanted Colo357 cells into the both flanks of immunocompromised mice. Upon establishment of tumor xenografts, mice were randomized in specific treatment and negative control groups. For siRNA delivery *in vivo*, polymeric nanoparticles based on polyethlyenimine (PEI) were used. By complexation of siRNAs with PEI, ~250 nm nanoscale polyplexes containing siRNA were obtained and used for intraperitoneal injection. Mice were injected 3x/week (10 µg siRNA/injection) and tumor volumes were regularly measured. To exclude non-specific PEI/siRNA effects, non-specific siRNA was complexed with PEI in the same way and served as negative control. Over ~3.5 weeks, a >15-fold increase in tumor volume was observed in the negative control group. In contrast, treatment with PEI-complexed specific siRNAs led to marked inhibition of tumor growth (Fig. 6A). Most prominent was the effect in the NRP2 knockdown group, with a >45% reduction in tumor size at day 25 after the initiation of treatment. To address the possibility of enhanced effects upon double knockdown, the experiment was repeated with a combination treatment protocol. More specifically, siNRP2 alone was compared with siNRP2 + siGIPC1 and with siNRP2 + siNRP1. While the tumor inhibition following only NRP2 knockdown was observed as before, there was no synergistic or additive inhibition of tumor growth in the combination groups.

Mice were sacrificed at the end of the treatment. Tumors were harvested and analyzed for target gene expression. Western blots revealed a ~50% reduction of NRP1 or NRP2 levels in the respective treatment group as compared to the negative control group (Fig. 6B). Western blots for GIPC1 from tissue samples were of insufficient technical quality and thus excluded from the analysis.

In a next step, we scored the immunohistochemical staining for NRP1, NRP2 and GIPC1 (Fig. 6C–H) by applying an immunoreactive score from 0–3 (Fig. 6I–K). As compared to the negative control treated group, NRP1 and NRP2 showed a prominent reduction of cytoplasmic and membranous staining in all tumor tissues of mice treated with PEI/siRNA nanoparticles against NRP1 (from 3 to 1 in the immune reactive score, with a 95% confidence interval of 2.421 to 2.879 in the control and 0.836 to 1.464 in the treatment group) or NRP2 (from 2 to 1 in the immunoreactive score, with a 95% confidence interval of 1.951 to 2.549 in the control group compared to 0.9925 to 1.607 in the treatment group). Likewise, upon treatment with PEI/siRNA nanoparticles containing siGIPC1 a significant downregulation was detected. The median of the immunoreactive score dropped significantly from 3 (95%CI: 2.311 to 2.789) in the untreated group to 1 (95% CI: 0.921 to 1.379) in the treated group. All cases show strong staining of peritumoral mouse macrophages, fibroblasts and vessels regardless of treatment. The quantitation also revealed that, in agreement with the immunoblot analysis, protein levels of those of the three candidate genes which were not targeted by the specific siRNA in the respective treatment group (e.g., NRP1 and NRP2 in case of siGIPC1) were not affected (Fig. 6I–K).

**Figure 7:**
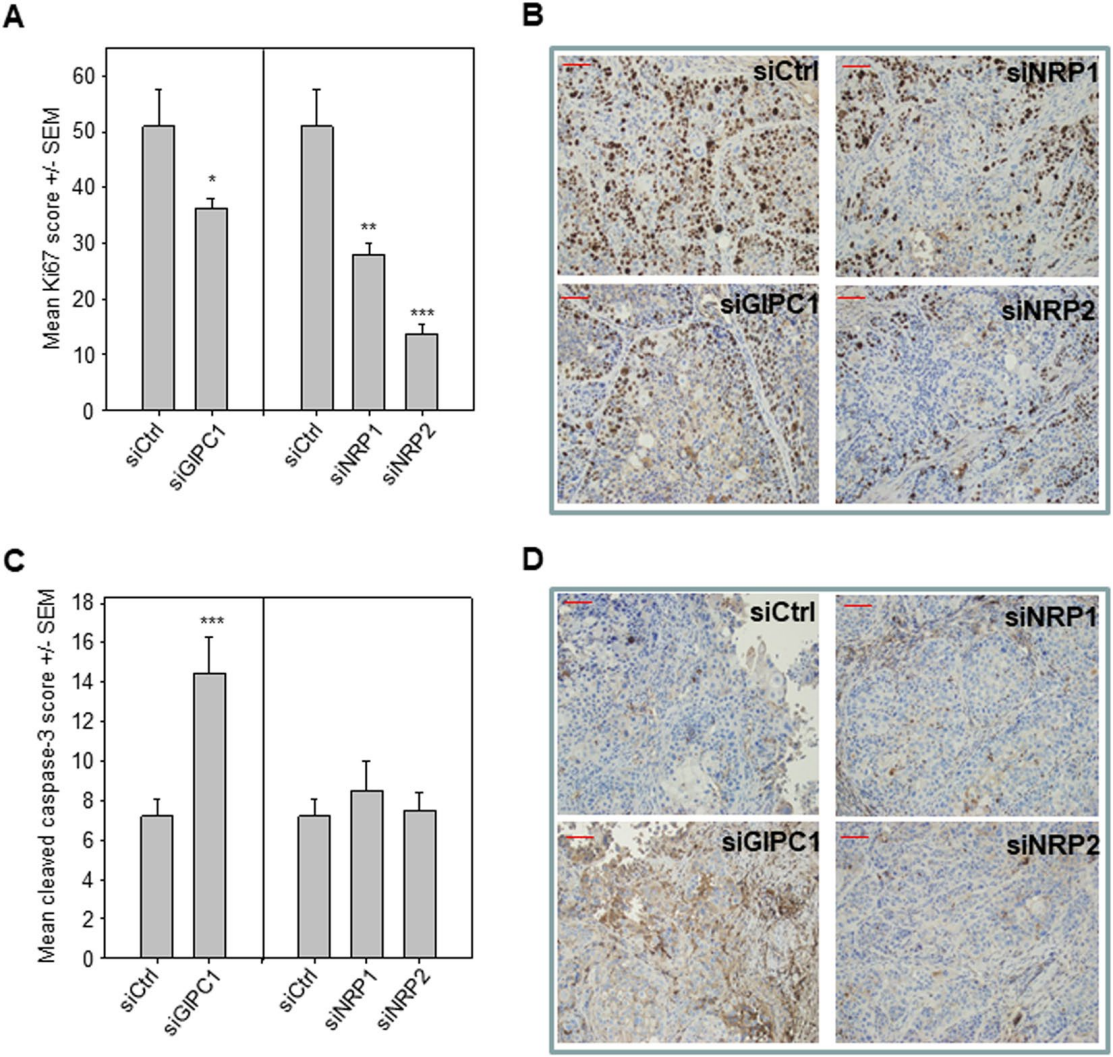
Quantitation of immunohistochemistry of tumor xenografts, stained with antibodies against the proliferation marker Ki-67 (**A**) and the apoptosis effector cleaved caspase-3 (**C**). (**B**) Representatives pictures of Ki67 staining in pancreatic cancer xenografts (200x). (**D**) Representative pictures of cleaved Caspase-3 staining of pancreaic cancer xenografts (200x). Bars: 50 µm.

To further analyze the underlying processes of tumor inhibition, we stained formalin fixed paraffin embedded sections of the tumors for markers of proliferation (Ki-67/mib-1) and apoptosis (cleaved caspase-3). Ki-67 staining revealed rapidly proliferating cancer cells throughout the whole tumor tissue in the control group. Proliferation was significantly reduced in the anti-NRP1 and anti-NRP2 treatment groups. Indeed, a 65% reduction of proliferation was observed in animals treated with NRP2 targeting nanoparticles (Fig. 7A,B). In contrast, a therapeutic knockdown of NRP1 or NRP2 did not affect apoptosis, whereas a significant 2-fold increase in cleaved caspase-3 immunoreactivity was found in the siGIPC1 group (Fig. 7C,D).

## Discussion

Ductal adenocarcinoma of the pancreas is an aggressive cancer type, with little treatment options. Therefore, there is a need for the development of novel therapeutic strategies. Based on previous studies demonstrating neuropilin overexpression in PDAC, which associate with poor survival and tumorigenic properties (see Introduction), we set out to comprehensively analyze tumor-inhibitory effects of siNRP1, siNRP2 and siGIPC1 either alone or in combination. SiRNA-mediated gene knockdown was explored because no specific inhibitors are currently commercially available for NRPs and GIPC1. It is also noteworthy that recently the first siRNA drug has received approval for patient treatment^28^ clearly indicating the therapeutic relevance and potential clinical usefulness of siRNAs for RNAi-mediated oncogene knockdown. The use of our previously developed polymeric nanoparticles for small RNA delivery^26,29,30^ (30 for review) addresses several issues of siRNAs as drugs, i.e., poor stability and cellular uptake, insufficient tissue penetration as well as rapid renal clearance and excretion. Thus, it allowed us to efficiently apply siRNA and analyze knockdown effects also *in vivo*, as demonstrated by substantial reduction of target gene levels in the tumors. It should be also noted that previous studies had demonstrated these nanoparticles based on low molecular weight PEI to be non-toxic, as demonstrated by the absence of any adverse effects (hepato- or renal toxicity, stimulation of the innate immune system, weight loss, or any other toxic effects), also after repeated application^26,31^.

Our *in vitro* studies demonstrated that some PDAC inhibitory effects are dependent on the cell line and the assay readout. The inhibitory effects of a given knockdown were found to be largely independent of initial expression levels, thus indicating that the degree of overexpression of the selected target gene is a poor predictor of its function. In most cases, absence of additive effects upon combined siRNA-mediated knockdown suggests that redundancies between the target genes are not a major issue when targeting the NRP axis. This is also confirmed by the absence of counter-upregulation of other NRP or GIPC1 as consequence of a single knockdown, which has been found in other oncogene families (Jenke *et al*., submitted; Gutsch and Aigner, unpublished). Concomitantly, no further tumor inhibition was observed when using siRNA combinations. For further enhancement of therapeutic efficacies, which is clearly warranted in the light of the only partial tumor inhibition, the combination with established chemotherapy or new therapeutic strategies will be promising, yet beyond the scope of this paper. In general, significant improvements have been made in patients with resectable disease who receive adjuvant chemotherapeutic treatment regimen^32^. In these patients the median survival is 28 months. Unfortunately, there has been very limited advancement in treating metastatic pancreatic cancer. The last landmark study for this group of patients was published in 1997 showing that gemcitabine is superior to 5-fluorouracil^33^. In other cancer entities there has also been a breakthrough of cancer immunotherapy including immune checkpoint inhibition (ICI). However, this success has not translated to the treatment of PDAC. Both common ICI therapies – the anti programmed death 1 (anti-PD-1) and the anti cytotoxic T-lmyphocyte-associated antigen 5 (anti-CTLA-4) – has demonstrated only limited success^34,35^.

Notably, in a recent study we found an important role of NRP2 during macrophage efferocytosis^36^, a mechanism that is important to clear debris from the tumor microenvironment. Ineffective efferocytosis leads to an enhanced immune response. In line with this and with our previous studies, one could imagine a combination therapy of NRP-dependent tumor cell and NRP2 dependent macrophage blockade in combination with conventional chemotherapy or ICI that might result in a significantly improved outcome in the treatment of advanced or metastasized PDAC. Indeed, conventional chemotherapy increases the cell debris which is then engulfed by macrophages via efferocytosis. NRP blockade leads to an inefficient efferocytosis that elicits immune responses. Consecutive ICI makes the tumor then more immunogenic.

While our findings of reduced anchorage-dependent and -independent growth after blockage of NRP1 are in line with, and further extend, previous studies, it should be noted that NRP1 has also been suggested to even suppress pancreatic cancer growth^37^. These different observations - pro-tumorigenic effects of NRP1 on the one side and tumor suppressing effects of NRP1 on the other - were attributed to the status of activating K-Ras mutations in pancreatic cancer^38^.

 Our results clearly indicate that depletion of GIPC1 by siRNA also shows a significant reduction in anchorage-dependent and -independent growth in pancreatic cancer. Compared to neuropilin knockdown, particularly profound effects were observed on cell cycle inhibition and induction of apoptosis. GIPC1 inhibition also significantly affected tumor growth *in vivo*. However, NRP2 silencing by PEI/siNRP2 nanoparticles showed the most substantial growth inhibition in the xenografts, indicating NRP2 blockage to be the most promising approach *in vivo*. This is interesting because our *in vitro* data mostly suggested that GIPC1 should have the largest effect. The analysis of the tumor tissues for markers of proliferation and apoptosis also indicates different mechanisms of tumor inhibition upon knockdown of the respective target gene. Taken together, this highlights the necessity to validate *in vitro* data in the *in vivo* situation. In this context, further studies using immunocompetent mice will additionally allow to address the role of NRP2 and its siRNA-mediated knockdown in tumor associated immune cells. We have previously shown that NRP2 derived effects in macrophages are essential for tumor growth^36^. Thus, beyond the tumor inhibition shown here, more profound effects may be anticipated in the immunocompetent context when knockdown strategies additionally benefit from affecting inflammatory cells in the tumor. Other limits of this study include that only one *in vivo* model was employed, not yet including a comparison with standard treatment (*in vitro* and *in vivo*). Clearly, further mechanistic studies are warranted to understand how cell genotype/phenotype results in different effects, independent from initial expression levels of the targets. In conclusion, NRPs and GIPC1 emerge as promising targets in PDAC treatment due to pleiotropic, non-redundant tumor-promoting effects. Their inhibition or, as in this study, knockdown affects proliferation/cell cycle and/or apoptosis/cell viability *in vitro* and *in vivo* and thus warrant further investigation with regard to effects on downstream signaling and therapeutic exploration in combination therapies.

## Supplementary information

Supplementary Information

## Data Availability

All the data are available within this manuscript.
