# Supplementary material for: Silencing of Neuropilins and GIPC1 in pancreatic ductal adenocarcinoma exerts multiple cellular and molecular antitumor effects
Source: Sci Rep. 2019 Oct 29;9:15471. doi: 10.1038/s41598-019-51881-8 (PMC6820541; doi:10.1038/s41598-019-51881-8)
Supplement: Supplementary file 1 — Supplementary Information [file 41598_2019_51881_MOESM1_ESM.pdf]

Supplementary Information

Silencing of Neuropilins and GIPC1 in pancreatic ductal adenocarcinoma exerts multiple cellular and molecular antitumor effects

Hannes Borchardt[1], Alexander Schulz[2], Kaustubh Datta[3], Michael Muders[4,5*] and Achim Aigner[1,5*]

[1] Rudolf-Boehm-Institute for Pharmacology and Toxicology, Clinical Pharmacology, Medical Faculty, University of Leipzig. Germany

[2] Institute of Pathology, University Hospital Carl Gustav Carus, University of Technology, Dresden, Germany

[3] Department of Biochemistry, University of Nebraska Medical Center, Omaha, Nebraska, USA

[4] Rudolf-Becker-Laboratory for Prostate Cancer Research, Institute of Pathology, University of Bonn Medical Center, Bonn, Germany

[5] Corresponding authors

[*]contributed equally as senior and corresponding authors

Suppl. Materials and Methods

Soft agar assay

For soft agar assays, cells were seeded in 12 well plates 24 h prior to transfection. 48 h after transfection, cells were trypsinized (Trypsin-EDTA; Biowest, Nuaillé, France) to obtain a single cell suspension and counted. For soft agar preparation, an autoclaved 2.4% agar solution (Carl Roth, Karlsruhe, Germany) and cell culture media were placed in a water bath to reach 42°C. A mixture of 12.5 mL agar solution, 1.25 mL MEM (10x) and 37.5 mL RPMI (10% FCS) were pipetted as bottom layer into a 6-well plate. For the top layer, 1 mL of a 6 x 10^4 cells/mL single cell suspension was mixed with 1.5 mL of the agar mixture, and 750 µL/well of this mixture was directly pipetted onto the bottom agar. Plates were incubated for 2 - 4 weeks and colonies > 50 µm in diameter were counted by 2 blinded investigators.

Colony formation assay

For colony formation assays, 1 x 10^3 cells in 2 ml culture medium were seeded in triplicates in a 6-well plate and cultivated for 7 - 9 days. Subsequently, cell culture medium was removed and cells were washed with 1 mL PBS, prior to adding 1 mL clonogenic reagent (0.5% methylene blue (w/v) in 50:50 (v/v) ethanol/water) to each well and incubating at room temperature for 45 min. Cells were washed at least 3 times with water prior to image acquisition under the microscope. Images were analyzed with the ImageJ software for quantitation of blue stained cells.

Scratch Assay

3.5 x 10^5 Panc89 cells / well were seeded in a 6-well plate 24 h prior to transfection. After transfection with 10 nM siRNA, the cells were further cultivated under standard conditions, and when they reached 100% confluency after 2 days, a scratch in the cell layer was introduced using a 200 µL tip. The cell layer was washed twice with PBS and once with cell culture medium to remove floating cells, prior to adding 2 mL cell culture medium to each well. The plate was placed in a microscopic chamber set to 37°C and 5% CO_2, and cell invasion into the scratches was documented automatically over 24 h at three different positions per scratch via a microscope, with pictures taken every 5 min. The percentage of scratch closure over time was quantitated using the ImageJ software and calculated relatively to t=0.

Caspase Assay and Annexin V staining

For caspase assays, cells were seeded and transfected as described above, and after 48 - 96 h 50 µL of the caspase-3/7-Glo reagent, diluted 1:10 or 1:5 in serum free medium depending on the cell line, was added to each well. After 1 h of incubation at room temperature in the dark, luminescence was measured by using a Fluostar Optima reader (BMG Labtec, Ortenberg, Germany). To normalize for different cell densities upon transfection, a WST-1 assay for quantitating viable cells was run in

parallel on the same plate. WST-1 was diluted 1:10 in serum free medium and 50 µL of this mixture was added to each well. After incubation at 37°C and 5% CO_2 for 1 h, the plates were measured at 450 nm in a DigiScan plate reader (Asys Hitech, Eugendorf, Austria). By calculating the ratio caspase activity / WST-1 signal, the caspase signal was adjusted to the cell density.

For Annexin V staining, cells were transfected as described above and harvested at different time points (48 - 96 h), prior to washing twice in ice cold PBS, resuspending at 1 x 10^6 cells/mL in 1x annexin binding buffer (10 mM HEPES, 140 mM NaCl, and 2.5 mM $CaCl_2$, pH 7.4) and addition of 5 µL of annexin V conjugate to each 100 µL cell suspension. During a 15 min incubation at room temperature, 5 µL propidium iodide (PI) solution (1 mg/mL) was diluted in 395 µL 1x annexin binding buffer per sample. 400 µL PI solution was added to each reaction tube and the cell suspension was incubated for further 5 min on ice in the dark. Samples were kept on ice until measurement in an Attune® Acoustic Focusing Cytometer (Thermo Scientific, Schwerte, Germany).

Cell Cycle Analysis

At 2 - 3 days post transfection, cells were treated for 14 - 20 h, depending on the cell line, with 25 ng/mL nocodazole in standard cell culture medium. Cells were harvested, washed once with PBS and fixed with 1 mL cold 70% ethanol for at least 1 h at -20°C. After fixation, the cells were spun down (1,000 rpm, 5 min) and washed once with ice-cold PBS, prior to resuspension in 50 µg/mL RNaseI solution in PBS and incubation for 1 h at 37°C. During incubation, 5 µL propidium iodide (PI; 1 mg/mL) were diluted in 345 µL PBS for each sample. The PI solution was added to the reaction tube and the cell suspension was incubated for further 5 min on ice in the dark. Samples were kept on ice until measured in an Attune® Acoustic Focusing Cytometer. The cell cycle distribution was determined using the Attune® Cytometric Software.

Western blotting

Lysates were prepared from cells in 6-well plates by adding 100 µl RIPA buffer and were homogenized by drawing the lysate up and down through a needle, using a 1 mL syringe. After centrifugation at 10,000 rpm for 10 min at 4°C, the supernatant was transferred to a new tube. Protein concentrations were measured using the Pierce BCA Protein Assay Kit (Thermo Scientific) according to the manufacturer's protocol and calculated from a bovine serum albumin standard curve.

For sodium dodecyl sulfate polyacrylamide gel electrophoresis (SDS-PAGE), samples containing 40µg protein were mixed with NuPAGE LDS sample buffer (Thermo Scientific) containing 5% β-mercaptoethanol (Sigma-Aldrich), heat denatured and loaded onto a precast NuPAGE 4-12% Bis-Tris gradient gel (Life Technologies) placed in an XCell SureLock Mini-Cell system (Invitrogen). After electrophoresis in NuPage MOPS buffer (Life Technologies) at 4°C for 3 hours at 120V, bands were

electroblotted onto a methanol activated PVDF membrane (Bio-Rad, Munich), using a PROTEAN Tetra Cell transfer system (Bio-Rad) for wet blotting, with NuPage blotting buffer (Life Technologies) containing 20% methanol. The blotting was performed at 80 V for 3 h or at 20 V overnight at 4°C. After blotting, the membrane was washed once with TBS-T (Tris-buffered saline (pH 7.4) + 0,05% Tween-20) and incubated for 1 h in 5 % milk powder in TBS for blocking of non-specific binding sites. Primary antibodies were diluted in 5 mL blocking solution as detailed in Suppl. Table 3 and used for membrane overnight incubation at 4°C. After washing three times with TBS-T, membranes were incubated in the secondary antibody dilution for 2 h at 4°C, followed by three additional washing steps. ECL substrate or, for higher sensitivity, Dura substrate (both Thermo Scientific) were used for visualization of protein bands. After exposure of X-ray films (GE Healthcare), bands were quantitated using the ImageJ software.

Supplementary Figure legends

Suppl. Fig. 1. (A) Expression levels are independent of cell confluency and FCS supplementation, and of housekeeping genes used for normalization. (B) Comparison of target gene knockdown mediated by different siRNAs, as determined by mRNA levels in Colo357 cells. (C) Knockdown of target gene mRNA mediated by optimal siRNAs, as determined in Colo357 cells. (D) Western blots demonstrating the reduction of target gene protein levels in AsPC1 (upper panel) and Colo357 cells (lower panel).

Suppl. Fig. 2. (A) Colony formation assays in Panc89 cells. (B) Caspase activation in Panc1 cells 72 h after knockdown. (C) Time-dependent scratch closure in a wound healing assay (Panc89 cells).

Suppl. Fig. 3. (A) Flow cytometry-based determination of apoptotic AsPC1 (left) or Colo357 cells (right) 72 h after transfection. (B) LDH release assay in Panc89 cells. (C) Anchorage-dependent proliferation assay in Colo357 cells. (D) Caspase activation in Colo357 cells 72 h after knockdown.

Attached also scans of original full immunoblots.

Supplementary Tables

Suppl. Table 1. Sequences of siRNAs used in this study

Suppl. Table 2. Sequences of primers used in this study

Suppl. Table 3. Antibodies used in this study

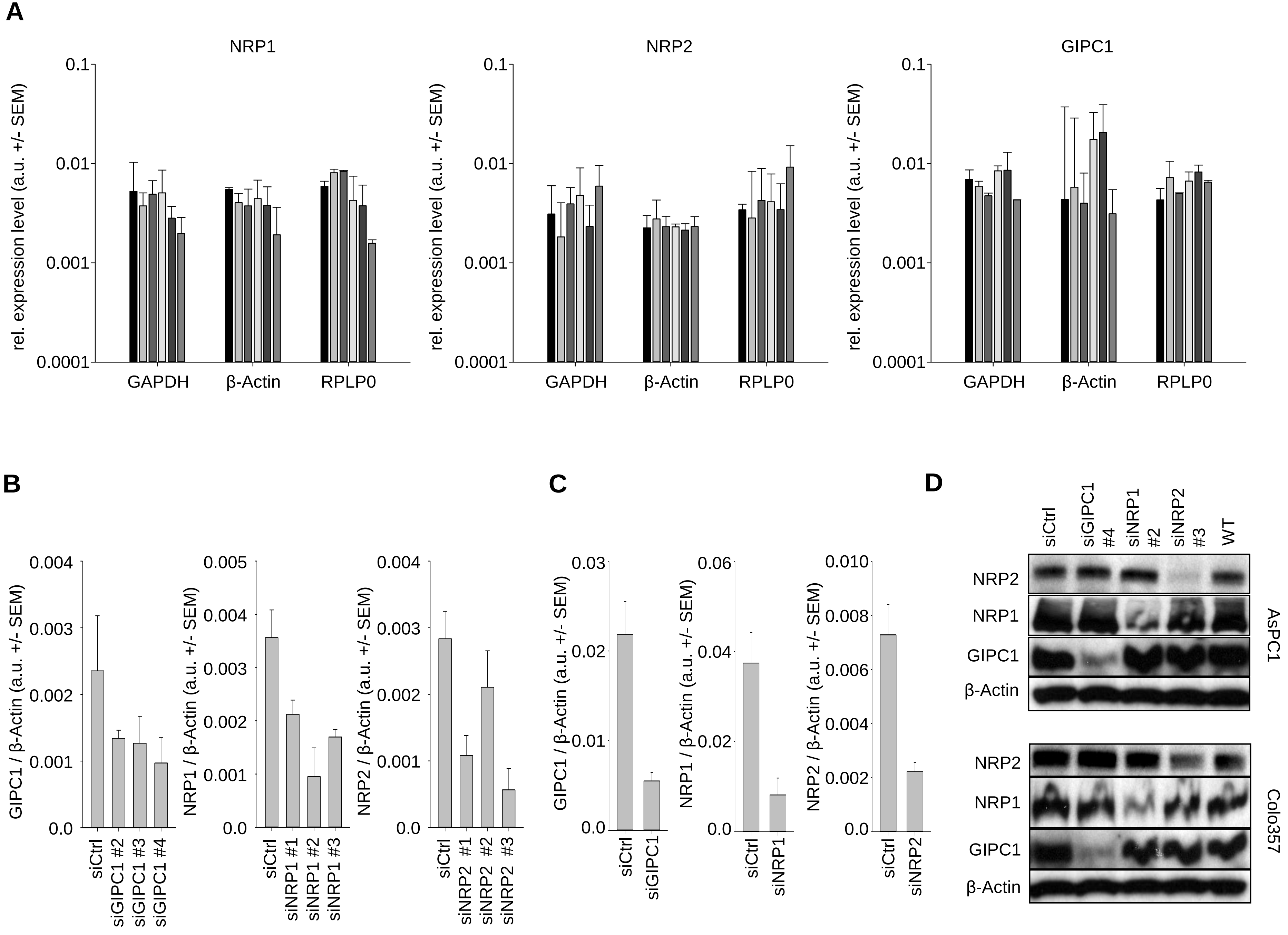

Borchardt et al. – Suppl. Figure 1

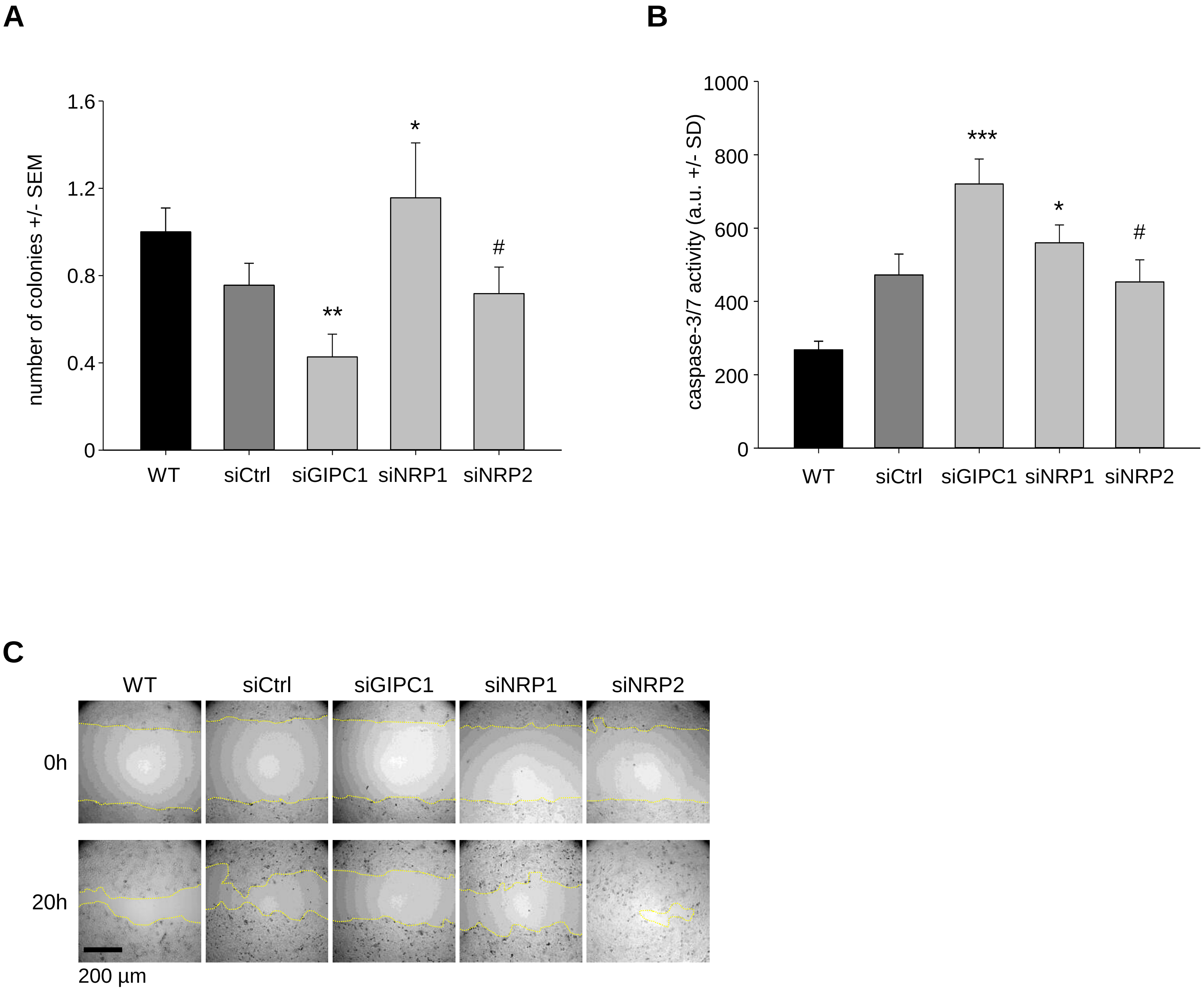

Borchardt et al. – Suppl. Figure 2

A

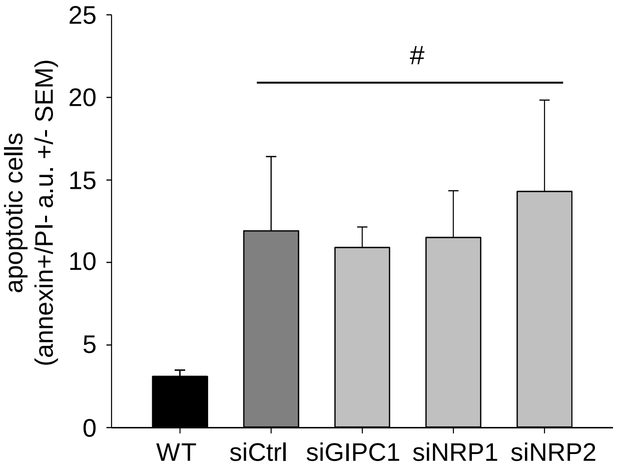

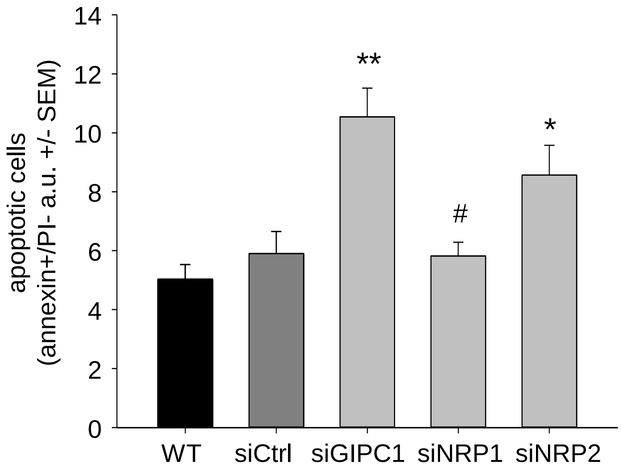

B

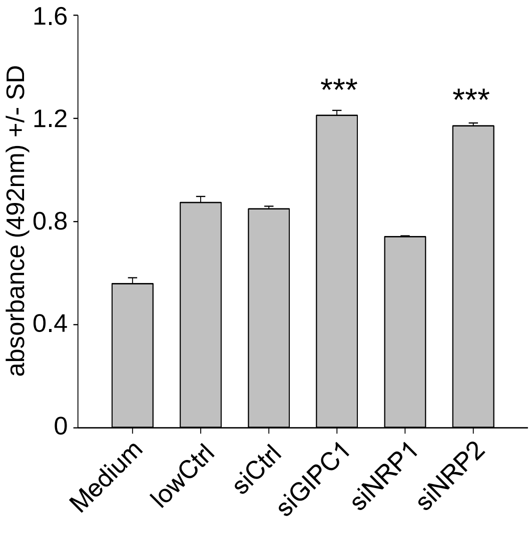

C

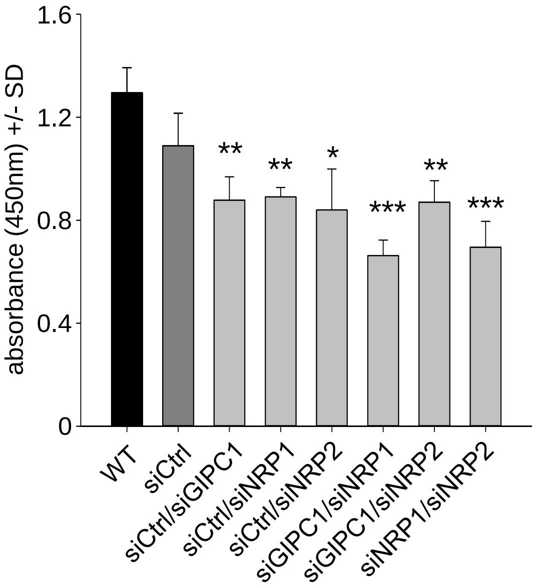

D

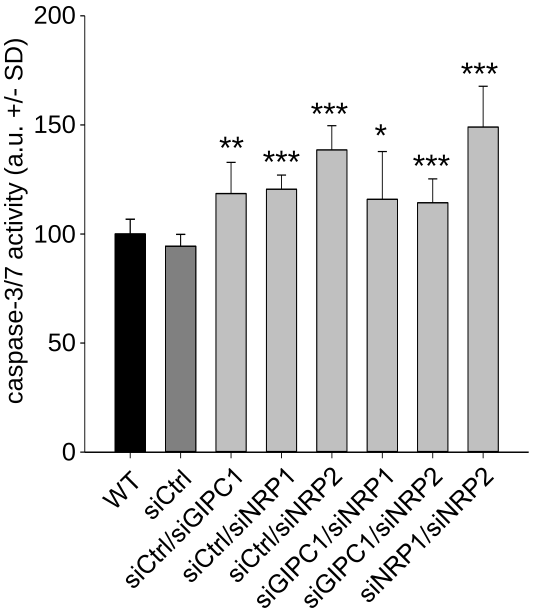

Borchardt et al. – Suppl. Figure 3

Suppl. Table 1. Sequences of siRNAs used in this study

Name	siRNA ID	Sense	Antisense
siGIPC1#2	256501	AUCUAUCUGCUGUUGGAAAtt	UUUCCAACAGCAGAUAGAUtc
siGIPC1#3	290345	CCUGCUGGAGAGUUACAUGtt	CAUGUAACUCUCCAGCAGGtc
siGIPC1#4	16786	GGCCAUUGAGAAGGUGGAUtt	AUCCACCUUCUCAAUGGCCtt
siNRP1#1	107269	GGCGAACUCUUUUGAGGGCtt	GCCCUCAAAAGACUUCGCCtt
siNRP1#2	4914	GGAUUUUCCAUACGUUAUGtt	CAUAACGUAUGGAAAAUCCtg
siNRP1#3	107268	GGAGAAAUUCAUUCUGACCtt	GGUCAGAAUGAAUUUCUCCtg
siNRP2#1	115159	GCCACUAGAGAACUUUCAGtt	CUGAAAGUUCUCUAGUGGCtc
siNRP2#2	107266	GGUUUGUUAGAAUCCGCCCtt	GGGCGGAUUCUAACAAACCtt
siNRP2#3	107264	GGUCGUUUGAAUUCCAAAGtt	CUUUGGAAUUCAAACGACCtc
siCtrl		CUUACGCUGAGUACUUCGAtt	UCGAAGUACUCAGCGUAAtt

Suppl. Table 2. Sequences of primers used in this study

Name	Forward-Sequence	Reverse-Sequence
GIPC1	ACGTCAAGGAGCTGTATGGC	CCTCCGACTTGAACACCTCC
NRP1	CCCCAAACCACTGATAACTCG	GTCCAGACACCATACCCAACAT
NRP2	AGGTGGACTGGAGCAATTCTTC	AGCTCTTTTCTTTGTCGGTCGA
β-Actin	CCAACCGCGAGAAGATGA	CCAGAGGCGTACAGGGATAG
GAPDH	AGCCACATCGCTCAGACAC	GCCCAATACGACCAAATC C
RPLP0	TCTACAACCCTGAAGTGCTTGAT	CAATCTGCAGACAGACACTGG

Suppl. Table 3. Antibodies used in this study

PRIMARY ANTIBODIES				
Antibody	Target Raised	Manufacturer	Application	Dilution
Human NRP2	Goat	R&D	WB<br>IF	1:400<br>1:250
Human NRP1	Rabbit	Abcam	WB	1:1000
Human GIPC1	Mouse	Santa Cruz	WB	1:500
Human b-Actin	Mouse	Abcam	WB	1:50000
SECONDARY ANTIBODIES				
Anti-goat, HRP-conjugated	Donkey	Dianova	WB	1:10000
Anti-mouse, HRP-conjugated	Goat	Abcam	WB	1:10000
Anti-rabbit, HRP-conjugated		Cell Signaling	WB	1:10000
ANTIBODIES FOR IMMUNOHISTOCHEMISTRY IN FFPE MATERIAL				
Neuropilin-1	Rabbit	Abcam	IHC-P	1:200 (microwaved antigen retrieval with TRIS/EDTA buffer, pH 9.0)
Neuropilin-2	Mouse	Santa Cruz	IHC-P	1:100 (microwaved antigen retrieval in TRIS/EDTA buffer, pH 6.0)
GIPC1	Goat	Abcam	IHC-P	10µg/ml (microwaved antigen retrieval in TRIS/EDTA, pH 9.0)
Cleaved Caspase-3	Rabbit	Zytomed	IHC-P	1:200 (heat induced antigen retrieval in citrate buffer, pH 6.0)
Ki67	Mouse (clone MIB-1)	Dako	IHC-P	Ready to use antibody

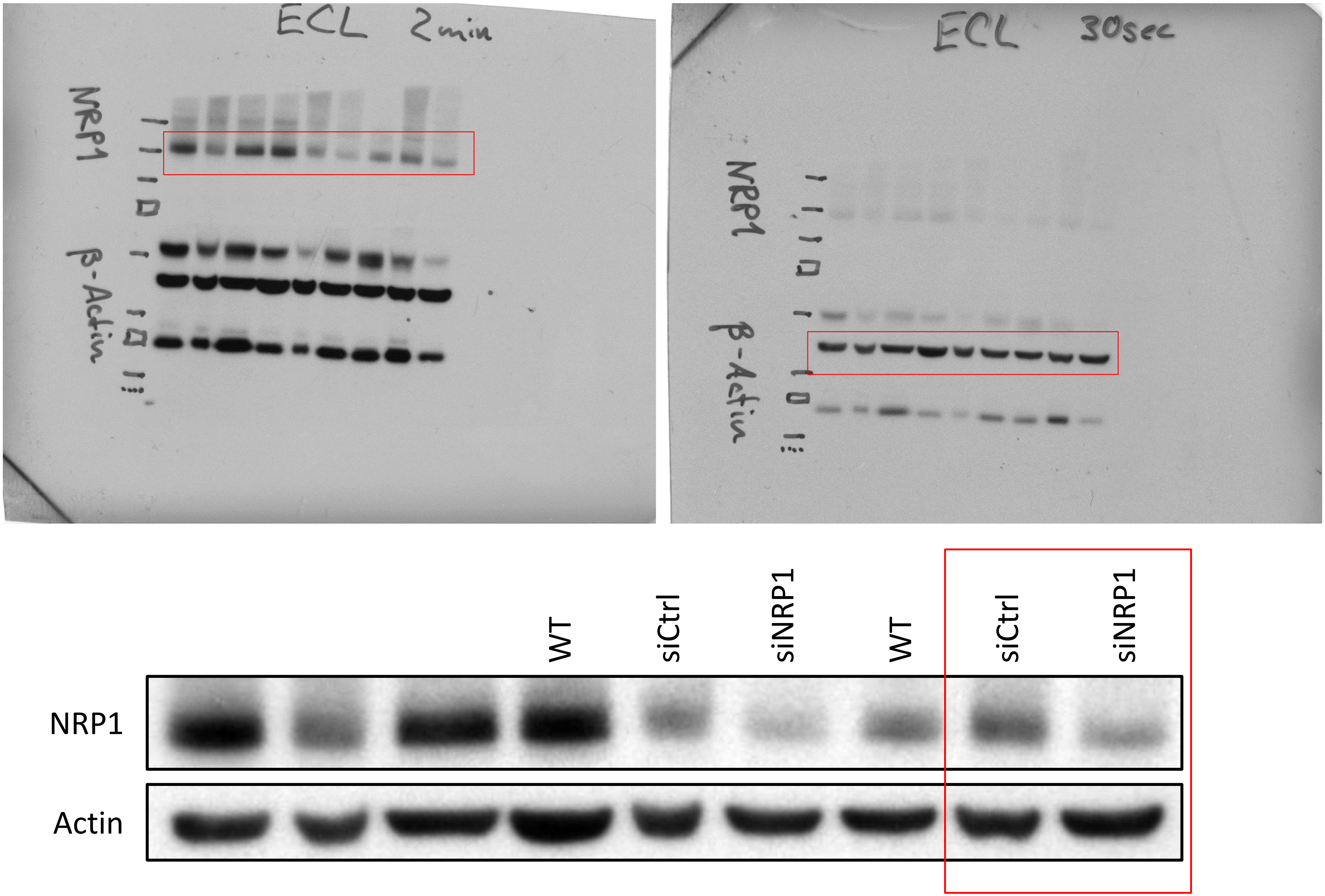
ECL 2min
NRP1
β-Actin
ECL 30sec
NRP1
β-Actin
WT
siCtrl
siNRP1
WT
siCtrl
siNRP1
NRP1
Actin

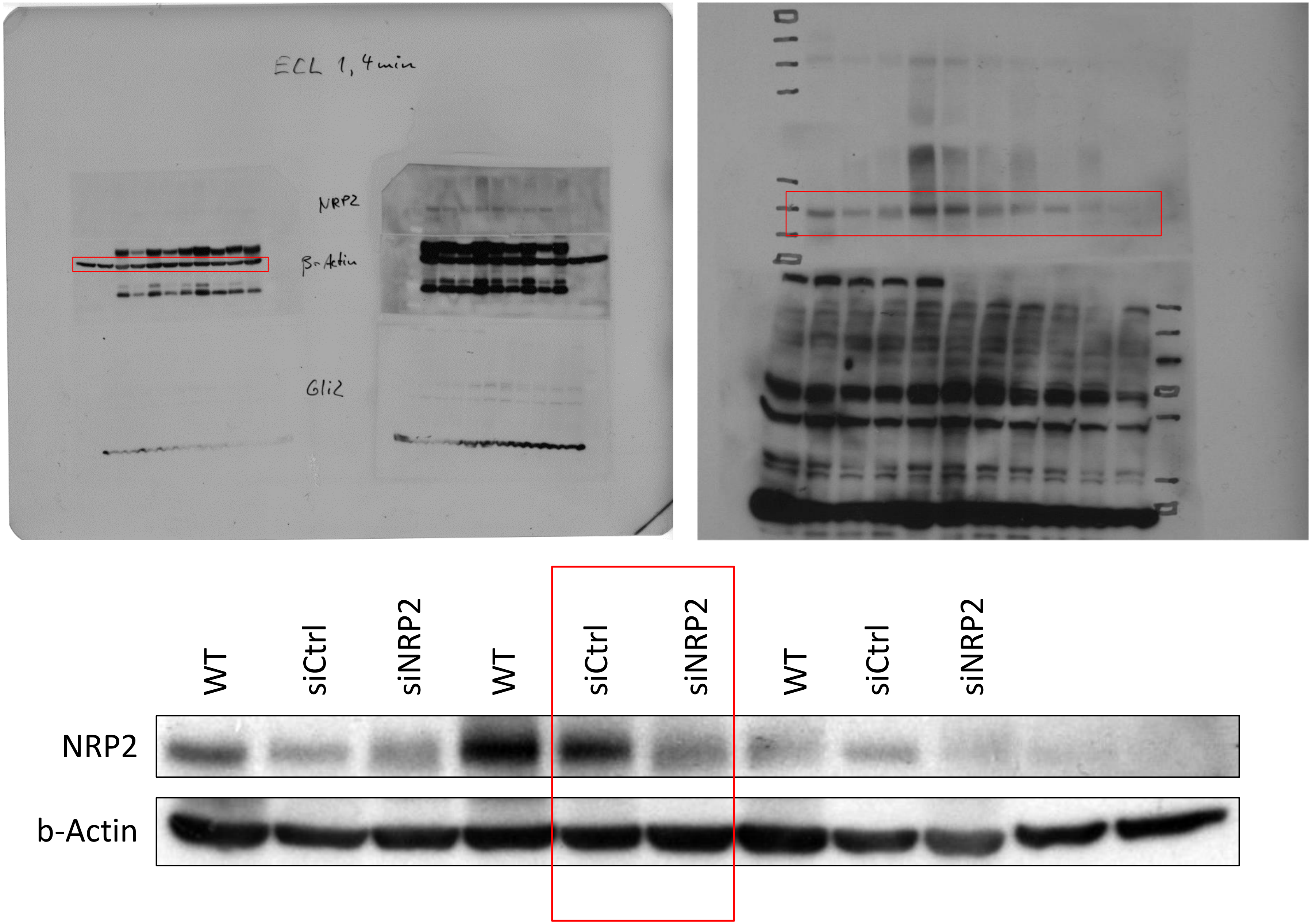

ECL 1, 4min
NRP2
β-Actin
Gli2
WT
siCtrl
siNRP2
WT
siCtrl
siNRP2
WT
siCtrl
siNRP2
NRP2
b-Actin
